# An Efficient CRISPR/Cas9 Platform for Rapidly Generating Simultaneous Mutagenesis of Multiple Gene Homoeologs in Allotetraploid Oilseed Rape

**DOI:** 10.3389/fpls.2018.00442

**Published:** 2018-04-20

**Authors:** Chao Li, Mengyu Hao, Wenxiang Wang, Hui Wang, Fan Chen, Wen Chu, Baohong Zhang, Desheng Mei, Hongtao Cheng, Qiong Hu

**Affiliations:** ^1^Oil Crops Research Institute of Chinese Academy of Agricultural Sciences, Key Laboratory of Biology and Genetic Improvement of Oil Crops, Ministry of Agriculture, Wuhan, China; ^2^Department of Biology, East Carolina University, Greenville, NC, United States

**Keywords:** allotetraploid, *Brassica napus*, CRISPR/Cas9, genome editing, *SPL3*, PAGE

## Abstract

With the rapid development of sequence specific nucleases (SSNs) for genome targeting, clustered regularly interspaced short palindromic repeats/CRISPR-associated protein 9 (CRISPR/Cas9) is now considered the most promising method for functional genetic researches, as well as genetic improvement in crop plants. However, the gene redundancy feature within the allotetraploid rapeseed genome is one of the major obstacles for simultaneous modification of different homologs in the first generation. In addition, large scale screening to identify mutated transgenic plants is very time-and labor-consuming using the conventional restriction enzyme-based approaches. In this study, a streamlined rapeseed CRISPR-Cas9 genome editing platform was developed through synthesizing a premade U6-26 driven sgRNA expression cassette and optimizing polyacrylamide gel electrophoresis (PAGE)-based screening approach. In our experiment, a sgRNA was constructed to target five rapeseed *SPL3* homologous gene copies, *BnSPL3*-A5/*BnSPL3*-A4/*BnSPL3*-C3/*BnSPL3*-C4/*BnSPL3*-Cnn. High-throughput sequencing analysis demonstrated that the editing frequency of CRISPR/Cas9-induced mutagenesis ranged from 96.8 to 100.0% in plants with obvious heteroduplexed PAGE bands, otherwise this proportion was only 0.00–60.8%. Consistent with those molecular analyses, *Bnspl3* mutants exhibited developmental delay phenotype in the first generation. In summary, our data suggest that this set of CRISPR/Cas9 platform is qualified for rapidly generating and identifying simultaneous mutagenesis of multiple gene homologs in allotetraploid rapeseed.

## Introduction

Oilseed rape is the predominant oil crop in China, northern Europe, and Canada, accounting for about 16% of the total global vegetable oil production (Hu et al., 2017; Woodfield et al., 2017). The modern widely cultivated oilseed rape is an allotetraploid species (*Brassica napus*; 2*n* = 38, AACC) that formed by polyploidization of two diploids ancestors, *Brassica oleracea* (genome C_O_C_O_) and *Brassica rapa* (genome A_r_A_r_) (Chalhoub et al., 2014; Liu S. et al., 2014). The genome merger and duplication caused by the polyploidy has been considered as a prominent evolutionary force for emergent properties, such as higher seed productivity and increased stress tolerance (Chalhoub et al., 2014; Liu S. et al., 2014). However, the allopolyploidization of *Brassica* genomes lead to multiple homologs of most genes in the oilseed rape genome compared with its diploid family species *Arabidopsis* (Chalhoub et al., 2014), which is one of the main obstacles for effectively generating simultaneous mutagenesis in multiple homoeologous sites by using conventional gene manipulation strategies.

Previous reports have demonstrated that high-frequency creation of DNA double-strand breaks (DSBs) using customizable sequence specific nucleases (SSNs) is an effective strategy for targeted mutagenesis at genomic sites of interests (Jackson, 2002; Lowder et al., 2016; Zhu et al., 2017). To date, several types of SSNs-mediated genome targeting technology have been developed to generate site-specific DSBs, including ZFN (zinc-finger nucleases), TALEN (transcription activator-like effector nucleases), and CRISPR/Cas9 (clustered regularly interspaced short palindromic repeats/CRISPR-associated) (Doyon et al., 2008; Zhang et al., 2010; Mali et al., 2013). Both ZFN and TALEN are artificial restriction enzymes that exert their cleavage functions by the combination of customizable ZFN or TALE DNA-binding domains (DBDs) with *Fok* I cleavage domains (Zhang et al., 2010, 2013; Schornack et al., 2013). Thus, ZFN and TALEN recognize their targeted DNA sequences through protein-DNA interactions. Although the mutagenic effectiveness of ZFN and TALEN has been reported in many plant species (Cantos et al., 2014; Wang et al., 2014; Char et al., 2015), the complicated and time-consuming DNA assembly process of ZFN/TALEN DNA-binding module is still a big challenge that hampers their extensive application.

In contrast, the RNA-guided CRISPR/Cas9 system mainly consists of three parts: Cas9 nuclease, a precursor CRISPR RNA (pre-crRNA) and a trans-activating crRNA (tracrRNA) (Jinek et al., 2012; Cong et al., 2013). Basically, the implement of CRISPR/Cas9-medited genome editing depends on three steps: (1) transcription of the programmable pre-crRNA, which encodes a unique RNA sequence complementary to targeted genome sequence; (2) Pre-crRNA was further processed into mature crRNA with association of tracrRNA; (3) DNA recognition and cleavage by crRNA-tracrRNA-Cas9 complex (Carroll, 2014). To simplify the DNA assembly procedure, crRNA and tracrRNA can be engineered into single guide RNA (sgRNA) transcript, which can guide Cas9 nuclease to recognize ~20 bp complementary DNA targets sequence that lies immediately 5′ of a protospacer-adjacent motif (PAM, NGG, or NAG) through Watson-Crick base pairing (Jinek et al., 2012; Cong et al., 2013). This feature extremely simplifies the assembly procedure of CRISPR/Cas9 genome editing components.

Currently, the RNA-guided CRISPR/Cas9 system has been rapidly adopted in a wide range of plant species, from model plant *Arabidopsis* and rice to crop plants with complex genomes, such as maize, tetraploid potato, tetraploid durum wheat, hexaploid bread wheat, and allotetraploid cotton (Jiang et al., 2013, 2014; Li et al., 2013, 2017; Upadhyay et al., 2013; Liang et al., 2014, 2017; Xing et al., 2014; Zhang et al., 2014, 2016; Wang et al., 2015; Andersson et al., 2017; Gao et al., 2017). Recently, CRISPR/Cas9 system was also employed to target multiple homologous sites of *B. napus* genes, including *BnALC, BnaRGA, BnaDA2, BnaFUL*, and *BnCLV* (Braatz et al., 2017; Yang et al., 2017).

However, it still lacks a set of simple and time-saving strategy to rapidly generate mutated plants in the first generation, which is essential to gene functional characterization and mutation screening at later generations. Basically, engineered CRISPR/Cas9 system consists of two modules, sgRNA and Cas9 protein expression cassettes, respectively. Previous study suggested that RNA polymerase III (Pol III) promoter *AtU6-26* has powerful capability to transcribe sgRNAs in dicot plants species (Li et al., 2007). Wang and his colleagues established a high efficient CRISPR/Cas9 system in cotton by employing a cotton U6-26 promoter (Wang et al., 2018). Currently, there are main two methods have been employed for identifying CRISPR/Cas9-induced mutations, PCR-restriction enzyme (PCR-RE) assay and T7 endonuclease 1 (*T7E1*) assay, respectively (Shan et al., 2014). However, both of those approaches are involved restriction enzyme digestion reaction, which are time and labor-consuming for large-scale screening of multiple homologs of rapeseed genes.

In this study, a premade U6-26 driven sgRNA expression cassette was employed in *B. napus* CRISPR/Cas9 system. Additionally, a PAGE-based screening approach was established for large scale identification of CRISPR/Cas9-induced mutagenesis. Deep sequencing and phenotypic analysis results demonstrated that the combination of U6-26-mediated CRISPR/Cas9 vector and PAGE-based mutation identification method is a time-saving and efficient strategy for rapidly generating mutated rapeseed plant in the first generation. In our *BnSPL3* case, this CRISPR/Cas9 system was successfully used in phenotypic identification in the first generation within four months, which shed the light on rapid rapeseed functional genomics study and the later stage of application.

## Results

### Experimental design and sgRNA expression cassette construction

**Figure 1:**
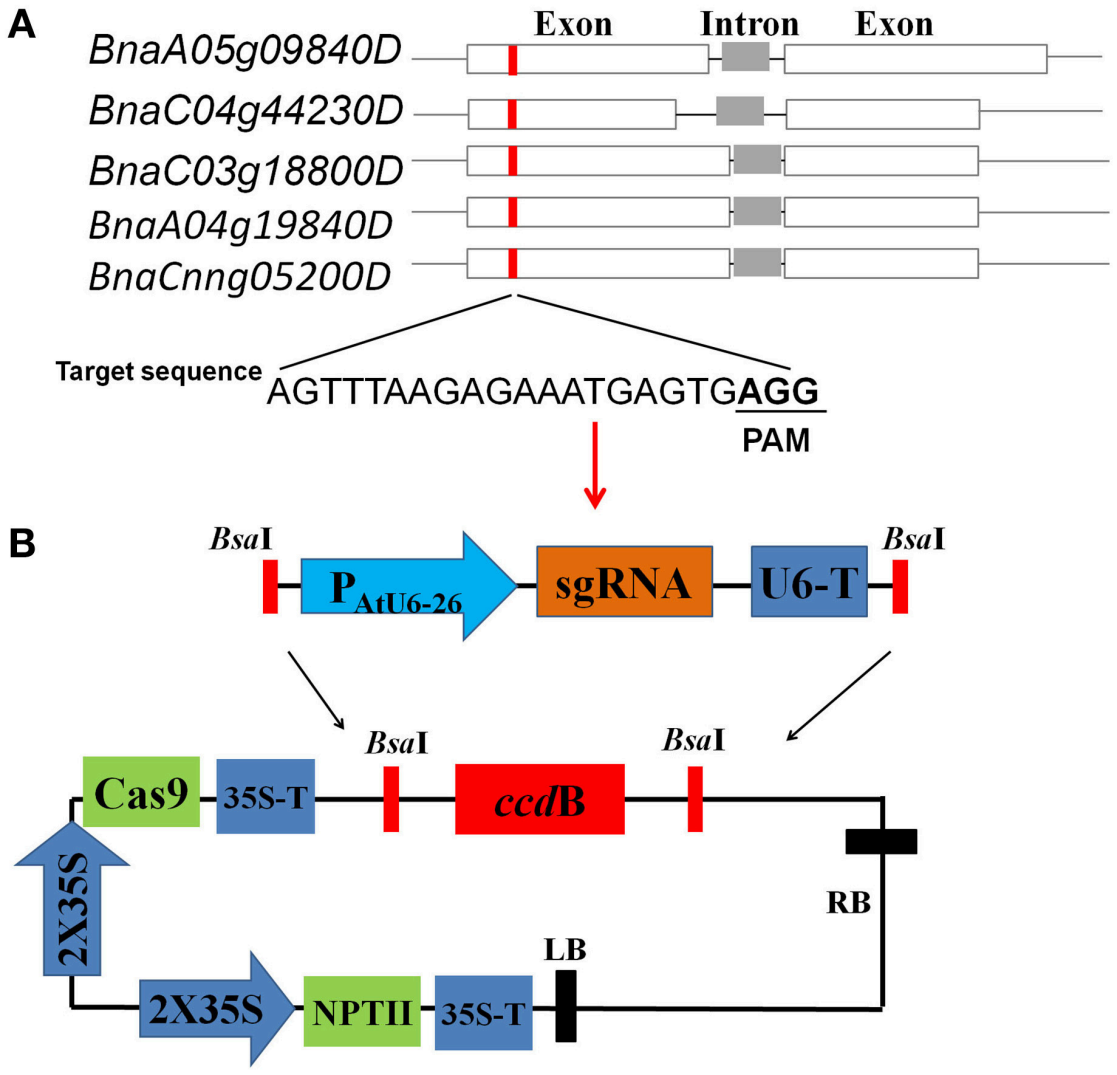
Target site design and CRISPR/Cas9 vector construction **(A)** Schematic description of target sites in the identical genomic regions of five rapeseed *SPL3* homoeologs, *BnaA05g09840D* (*BnSPL3A*), *BnaC03g18800D* (*BnSPL3B*), *BnaC04g44230D* (*BnSPL3C*), *BnaA04g19840D* (termed *SPL3D*), and *BnaCnng05200D* (termed *SPL3E*). **(B)** Diagrams illustrating the assembly procedure of CRISPR/Cas9 system. Based on PCR-based assembly strategy, the synthesized sgRNA sequence was rapidly assembled into a premade AtU6-26 promoter-driven expression module (upper panel). The expression module containing designed sgRNA sequence was inserted into destination vector pYLCRISPR/Cas9P35S-N through Golden Gate reaction (lower panel).

Given that RNA polymerase III (Pol III) promoters *AtU6-26* have powerful capability to transcribe sgRNAs in dicot plants species (Li et al., 2007), we premade a sgRNA expression module driven by *AtU6-26* promoter, termed AtU6-26-sgRNA. As shown in Figure 1B, AtU6-26-sgRNA expression module flanked by two *Bsa*I-cutting sites, which can be used for Golden Gate ligation reactions in the following step. In our experiment, five days is sufficient to complete all the process of CRISPR/Cas9 vector construction, from sgRNA design to vector assembly.

To evaluate the editing efficacy of this set of AtU6-26-mediated CRISPR/Cas9 system, five rapeseed orthologs of *Arabidopsis SPL3, BnaA05g09840D* (termed *SPL3A*), *BnaC03g18800D* (termed *SPL3B*), *BnaC04g44230D* (termed *SPL3C*), *BnaA04g19840D* (termed *SPL3D*), and *BnaCnng05200D* (termed *SPL3E*) were identified as candidate sequences for designing sgRNAs (Figure 1A, Cheng et al., 2016). Sequence analysis showed that all of five *SPL3* homoeologs are composed of two exons and one intron (Figure 1A). Through comparing the genome sequence similarity of rapeseed *SPL3*A/B/C/D/E, a 22-bp target sequence located in their exon region was selected for constructing CRISPR/Cas9 vectors (Figure 1A).

### Rapeseed transformation and rapid screening of CRISPR/Cas9-induced mutations in *BnSPL3* homoeologs

**Figure 2:**
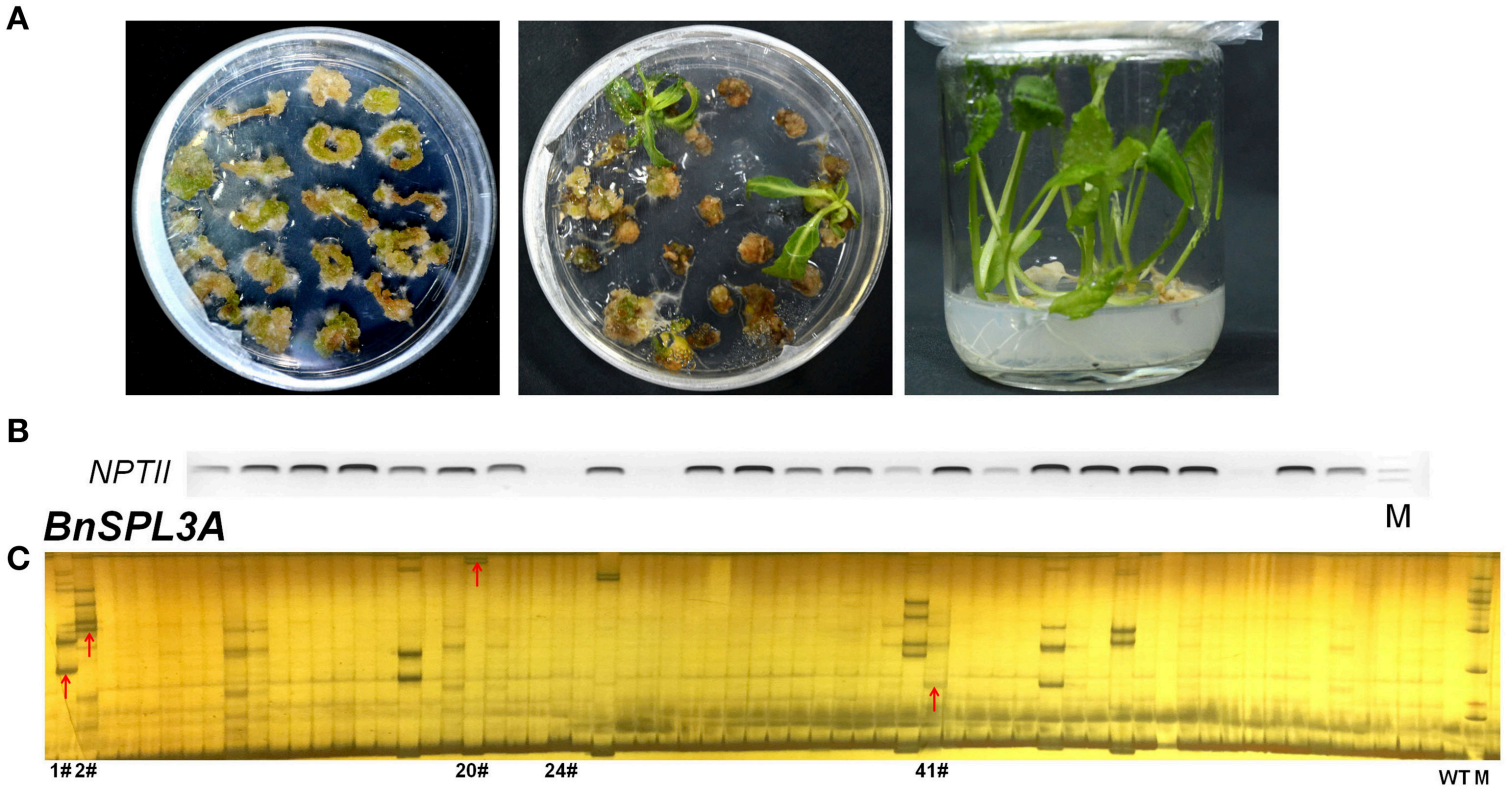
Rapeseed transformation and PAGE-based rapid screening of CRISPR/Cas9-induced mutagenesis in *BnSPL3* genomic sites. **(A)** Excised rapeseed hypocotyl segments were used as explants for *A. tumefaciens*-mediated transformation. **(B)** Identification of positive transgenic events by specifically amplifying *NPTII* gene. **(C)** Large scale screening of CRISPR/Cas9-induced mutagenesis in the genomic sites of *BnSPL3A*.

The selection of antibiotic marker is a key factor for plant regeneration. Based on our previous experiment, kanamycin is the most efficient selective marker for rapeseed regeneration compared with hygromycin and herbicide. Kanamycin always takes 2.5–3 months for regenerating transgenic plants, whereas hygromycin and herbicide need 4 and 10 months, respectively. As shown in Figures 2A,B, a lot of transformed rapeseed plantlets were regenerated from selective medium and the majority of them were positive transgenic plants.

Polyacrylamide gel electrophoresis (PAGE) method has been extensively used in high-throughput analysis of genotypic variation in breeding populations. To apply PAGE-based method in rapid screening of putative *SPL3* mutants induced by CRISPR/Cas9, five pairs of primers were used to specifically amplify designed mutation region of *BnSPL3A/B/C/D/E* genomic region. Through optimizing the procedure of PAGE assay, the heteroduplexed DNA fragment was successfully detected in many independent transgenic lines, such as #1, #2, #20, #41 (Figure 2C and Figures S1A–D) for all the five *BnSPL3* homologous sites, which suggests that CRISPR/Cas9-induced mutagenesis occurred in those designed genomic regions.

### High-throughput sequencing evaluation of CRISPR/Cas9-induced mutagenesis in *BnSPL3* homoeologs

Based on PAGE-based genotyping results, five transgenic plants (1#, 2#, 20#, 24#, 41#) were selected for further high-throughput sequencing analysis of the mutation efficacy of this set of CRISPR/Cas9 system. Among those five plants, excepting transgenic line 24#, all the rest plants displayed obvious heteroduplexed DNA bands in PAGE-based assays (Figure 2C and Figures S1A–D). Thus, the editing events of plant samples 24# was speculated to be very low compared with the others if our PAGE-based mutation screening method is accurate.

**Figure 3:**
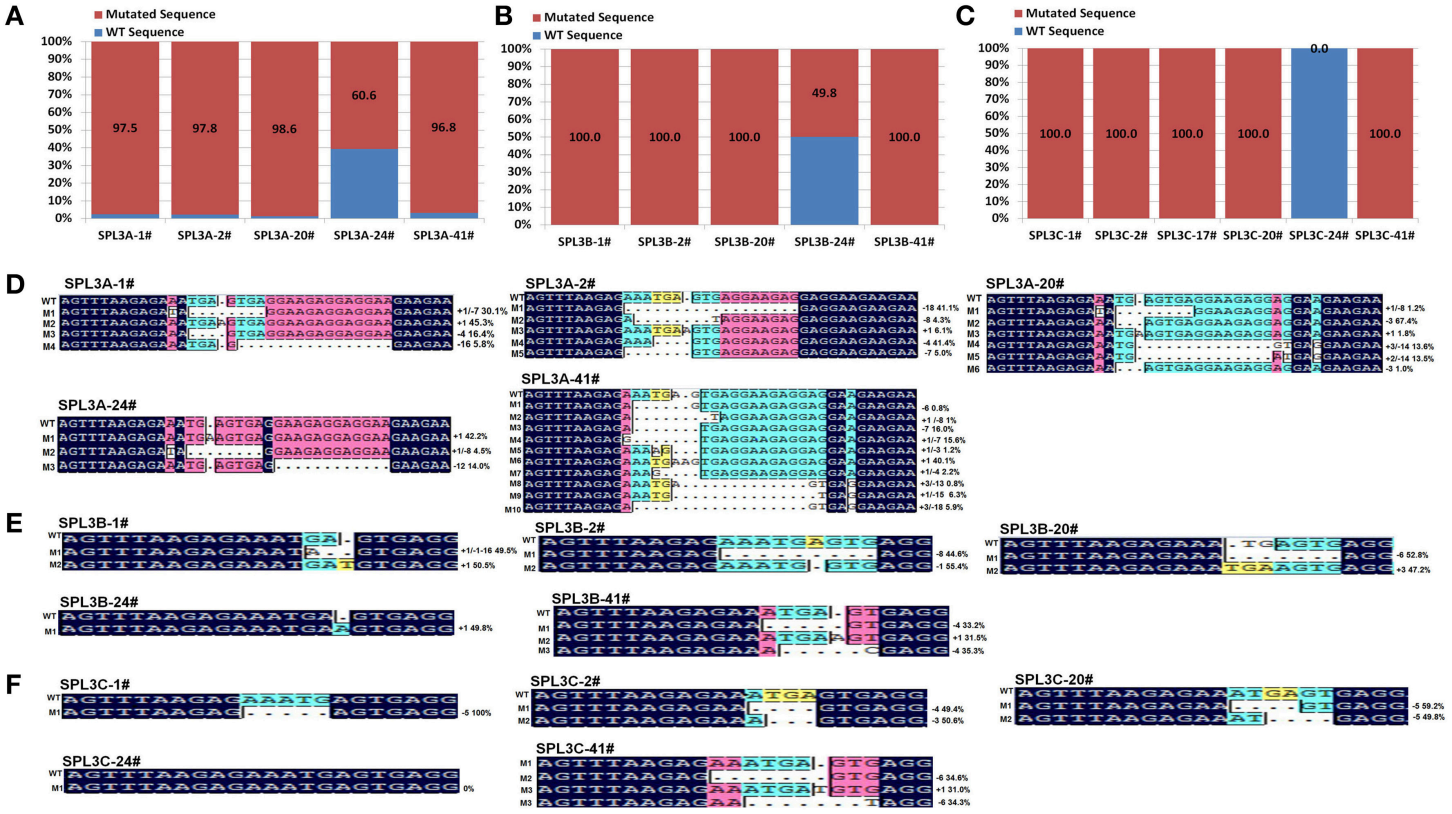
High-throughput sequencing analysis of CRISPR/Cas9-Induced mutagenesis in three *BnSPL3* homoeologs **(A–C)**, statistical analysis of editing frequency occurred in rapeseed *SPL3A, SPL3B*, and *SPL3C* genomic sites, respectively. Rapeseed sample 1#, 2#, 20#, 24#, 41# were from independent transgenic events. Excepting transgenic line 24#, all the rest samples display obvious heteroduplexed DNA bands in PAGE-based assays. **(D–F)** Sequences alignment of different mutation types identified from high-throughput sequencing analysis.

To test this hypothesis, 15 PCR products, covering the designed *BnSPL3A/B/C* mutation region of those selected transgenic plants, were purified for next generation sequencing. Finally, over 5,000,000 reads were obtained from each PCR samples. The reads were then used to match with wild type *BnSPL3A/B/C* genomic sequence and calculated the proportion of CRISPR/Cas9-caused mutagenesis. As expected, statistic data demonstrated that the mutation proportion of those four plants is estimated to range from 96.8 to 100% in *BnSPL3* A05 genomic site and 100% in both *BnSPL3* C03 and C04 genomic sites, whereas editing frequency of plant 24# is only 60.6, 49.8, and 0.0% in *BnSPL3* A05, C03, and C04 genomic sites, respectively (Figures 3A–C). Thus, PAGE-based screening method is a very effective strategy for rapid obtaining mutated rapeseed plants in the first generation.

As shown in Figures 3D–F, the majority of indel mutation were +1bp insertion and –1bp/–3/–4/–5/–7/–8 deletion. Although mosaic mutations occurred in many examined *BnSPL3* genomic sites, several plants displayed less mutation types in three *BnSPL3* genomic sites, such as the mutation type of sample SPL3-1# was only 3, 2, and 1 in *BnSPL3* A05, C03, and C04 genomic sites, respectively, which facilitate the process of identifying of homozygous mutation in the later generation. Taken all together, those results demonstrated that it is an effective strategy to rapidly generate mutated rapeseed plants in the first generation by combining of AtU6-26-mediated CRISPR/Cas9 genome editing system and PAGE-based screening method.

### *BnSPL3* is a key regulator in vegetative-to-reproductive phase transition

**Figure 4:**
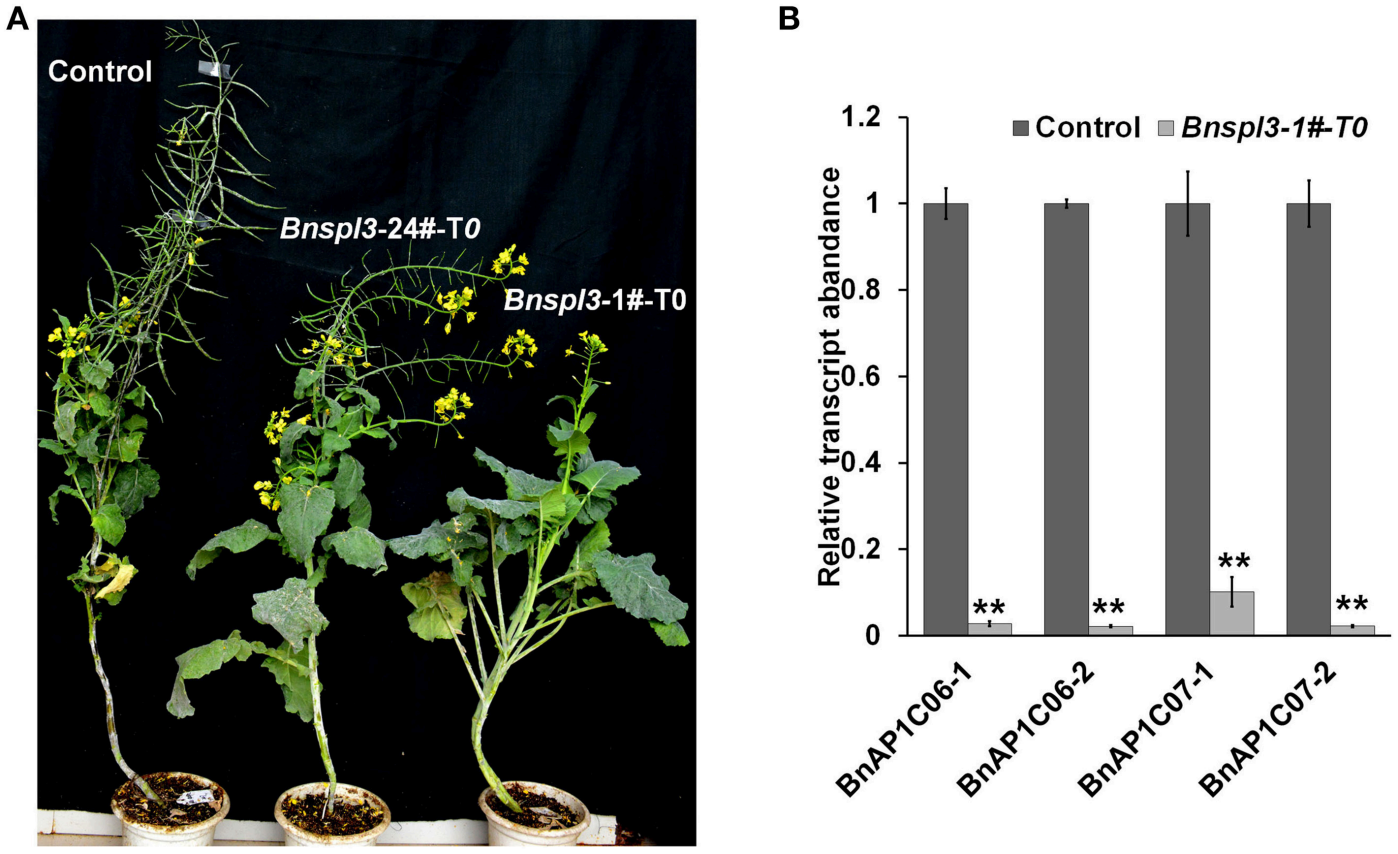
Morphological phenotypes of a *BnSPL3* transgenic line **(A)**, *Bnspl3* mutant exhibit severe developmental delay in the first generation. Control plant was regenerated at the same time with *Bnspl3* mutant plant. The editing frequency of *Bnspl3*-1#-T_0_ line is much higher than *Bnspl3*-24#-T_0_ line based on deep sequencing data. **(B)**, qRT-PCR analysis of four rapeseed homologs of *AtAP1*. Values are means ± SD; *n* = 3. Statistical analyses were performed using Student's *t*-test, ^**^ means *P* < 0.01.

To determine whether this set of CRISPR/Cas9 system can be used for gene functional characterization in T_0_ generation, a negative transgenic plant was used as a control to observe the phenotypic differences under normal growth conditions. As shown in Figure 4A, *Bnspl3* mutant exhibited different degree of developmental delay phenotype depended on the CRISPR/Cas9-induced editing efficacy under normal growth condition, which suggests that *BnSPL3* is probably implicated in the regulation of developmental phase transition, similar to its orthologs in Arabidopsis (Jung et al., 2012).

In Arabidopsis, *AtSPL3* were known as a direct upstream activator of *APETALA1*(*AP1*) (Yamaguchi et al., 2009). To examine whether the mutagenesis occurred in *BnSPL3* genomic sites could negatively affect rapeseed *AP1* transcript, the expressions of four rapeseed orthologs of *AtAP1, BnAP1C06-1*/*BnAP1C06-2*/*BnAP1C07-1*/*BnAP1C07-2*, were analyzed by qRT-PCR method. Similarly, all of those four *BnAP1* genes were strongly suppressed in plant sample 1# compared with control (Figure 4B). Thus, *BnSPL3* is likely to act upstream of *BnAP1* to regulate the developmental phase transition.

### Inheritance of CRISPR/Cas9-induced mutagenesis

**Figure 5:**
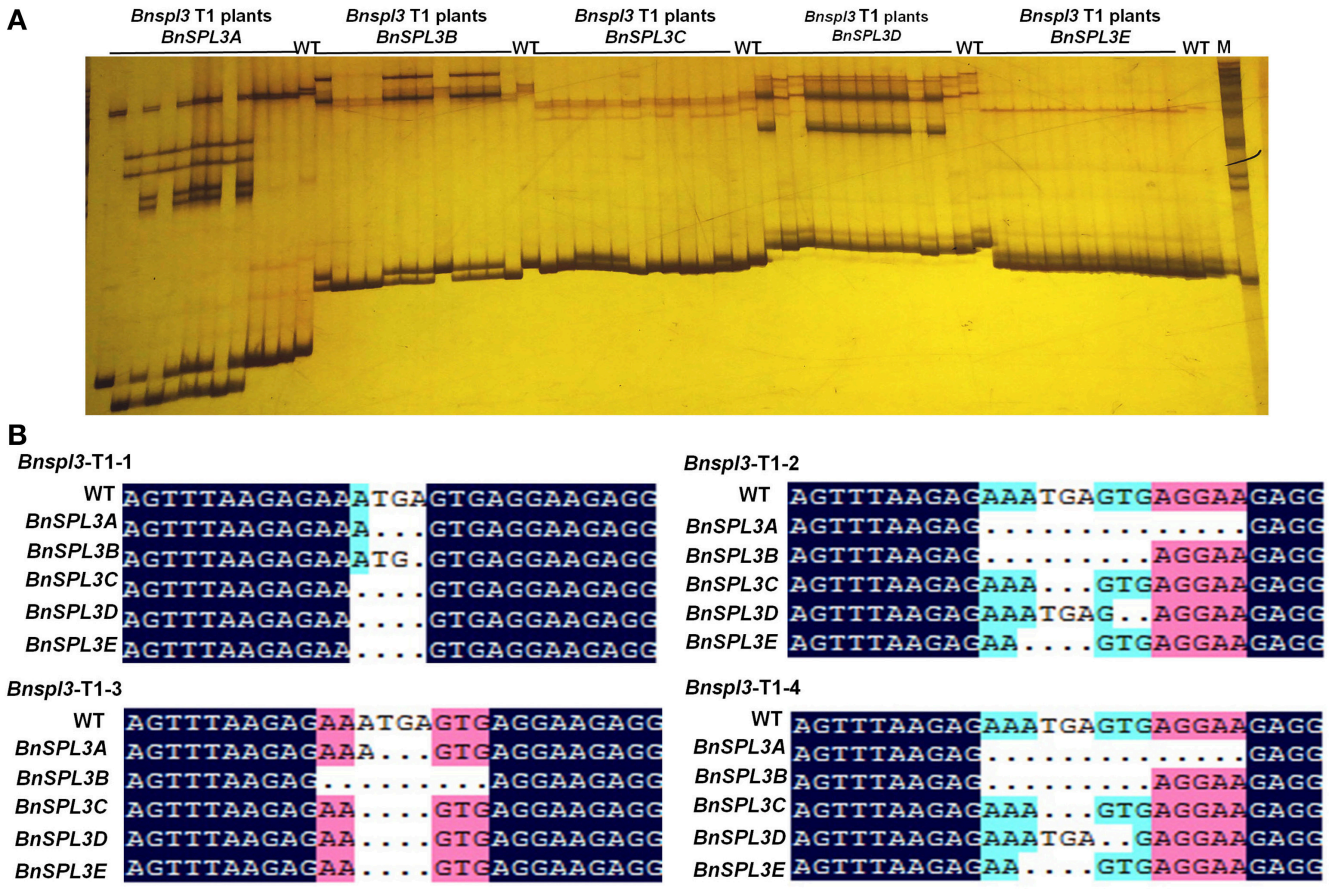
Evaluation of the inheritance of CRISPR/Cas9-induced mutagenesis in T1 generation. **(A)**, PAGE-based genotyping of the *Bnspl3* T1 generation plants at five *BnSPL*3 homologous sites. **(B)**, Sanger sequencing analysis of the mutation type in *Bnspl3* T1 generation plants.

To evaluate the inheritance of CRISPR/Cas9-induced mutagenesis in T1 generation, 12 T1 plants from genetic family #2 were used for PAGE-based genotyping analysis. As shown in Figure 5A, the heteroduplexed DNA bands were detected in the most of *Bnspl3* T1 progenies at all 5 homoeologous genomic sites. To further confirm this result, four *Bnspl3* T1 plants, *Bnspl3*-T1-1, *Bnspl3*-T1-2, *Bnspl3*-T1-3, and *Bnspl3*-T1-4, were used for PCR product Sanger sequencing analysis. Sanger sequencing results suggested that all of four tested T1 generation plants carried the mutated alleles at five *BnSPL3* homoeologous sites (Figure 5B). Take together, those molecular results demonstrated that CRISPR/Cas9-induced mutations can be transmitted to their rapeseed progenies.

## Discussion

With the rapid development of CRISPR/Cas9 genome editing technology, conventional genetic engineering breeding was undergoing a revolutionary change in the past few years. Recently, CRISPR/Cas9 has been proven to be effective for simultaneously modifying multiple homoeologous genomic sites of rapeseed genes, such as two rapeseed *ALC* homoeologs and four rapeseed *BnaRGA* homoeologs (Braatz et al., 2017; Yang et al., 2017). However, there still existing several open questions that hamper the extensive applications of CRISPR/Cas9 genome editing. In the majority of cases, each rapeseed transformation generated several dozens or hundreds of positive transgenic plants (Yang et al., 2017). For example, 233 transgenic plants was generated for screening of CRISPR/Cas9-induced mutagenesis at the alleles in Yang's paper (Yang et al., 2017). For our *BnSPL3* case, 72 independent positive transgenic plants were obtained after only one genetic transformation, and each transgenic plant had five homologous genome sites need to be evaluated for identifying simultaneous mutagenesis of multiple homoeologs in the first generation. Basically, two key factors determined whether we can rapidly obtain multiple homologs mutated plants: (1), the editing efficacy of CRISPR/Cas9 vectors; (2), efficient and time-saving mutation screening method.

CRISPR/Cas9 genome editing vector was composed of two main functional parts, one is 35S/ubiquitin promoter-driven Cas9 expression cassette, and the other one is RNA polymerase III (Pol III) promoter-driven gRNA expression cassette. Recent studies suggest that the transcriptional activity of Pol III promoter is a key limiting factor for determining the mutation efficacy of CRISPR/Cas9 system (Lowder et al., 2016; Tang et al., 2017; Wang et al., 2018). Previous study suggested that AtU6-26 have the highest transcriptional activity in model dicot plant *Arabidopsis* compared with AtU6-1 and AtU6-29 (Li et al., 2007). Thus, the core promoter element of AtU6-26 was introduced to construct sgRNA expression module. Our deep sequencing analysis results demonstrated that the proportion of CRISPR/Cas9-induced mutagenesis ranged from 96.8 to 100.0% by using AtU6-26 promoter, which is comparable with previous reports in *B. napus* (Braatz et al., 2017). Given that the high-efficacy of AtU6-26 promoter in rapeseed genome editing, we believed that AtU6-26 promoter is qualified for mediating CRISPR/Cas9 genome editing system in *B. napus* genome.

In addition, the selective marker is also crucial for plant regeneration and transformation. By comparing three selective markers, kanamycin, hygromycin and herbicide, we found that kanamycin takes 2.5–3 months for rapeseed regeneration, whereas herbicide need to take almost 10 months. Consistence with this result, Braatz et al. (2017) reported that 9–11 months of tissue culture period was necessary if herbicide selective marker was used in CRISPR/Cas9 vectors. Thus, kanamycin is probably the most suitable for rapid regeneration of rapeseed transgenic plants and has been employed in this CRISPR/Cas9 genome editing system.

To establish a simple and high-efficiency mutation identification approach, PAGE-based genotyping method was used to analyze CRISPR/Cas9-induced mutagenesis at five designed *BnSPL3* genomic sites. As expected, heteroduplex DNA bands were detected in many independent transgenic lines, this suggests that CRISPR/Cas9-induced mutation events probably occurred in those transgenic plants. Consistent with the PAGE screening results, high-throughput sequencing data showed that the editing frequency of the transgenic plant with obvious heteroduplexed DNA bands ranges from 96.8 to 100%, whereas the transgenic plant with imperceptible heteroduplexed DNA bands ranges from 0 to 60.6%. Thus, PAGE-based mutation screening technology is a time-saving and high-efficient approach to rapid identification of CRISPR/Cas9-induced mutagenesis in *B. napus* genome, which is especially suitable for large scale screening of CRISPR/Cas9-induced mutation population. In addition, the high-throughput sequencing results also suggest that AtU6-26-mediated CRISPR/Cas9 genome editing system is qualified for simultaneously generating mutation in multiple rapeseed gene homoeologs.

Although PAGE-based genotyping method has obvious advantages in terms of time efficiency, it still has its limitation of application, such as it is very hard to detect small nucleotide indels or substitution in homozygous mutation plants (Zhu et al., 2014). Previous report suggests that PAGE gel is capable of detecting the different at single nucleotide level based on the conformational difference of single-stranded DNA (Shirasawa et al., 2004). Thus, PAGE-based genotyping methods have the potential to be modified for screening of small nucleotide mutation induced by CRISPR/Cas9. In addition, several experimental conditions may affect the mutation type analysis, including the specificity of primers, the concentration and homogeneity of PAGE gel and the voltage of gel electrophoresis. Thus, PAGE-based mutation screening approaches can be employed for the first step identification of large scale mutation sites. After that, those candidate plants will be selected for further confirmation of the mutation type by sequencing methods.

Previous study demonstrated that *SPL3* is key floral activator acting upstream of *AP1* in *Arabidopsis* (Yamaguchi et al., 2009). *Bnspl3* mutant exhibited severe delay in the transition from vegetative to reproductive phase in the first generation. In addition, qRT-PCR analysis results also suggest that four rapeseed homoeologs of *AtAP1* were strongly suppressed in *Bnspl3* mutant compared with control plant.

Recently, CRISPR/Cas9 technology was employed to perform large-scale functional characterization in T_0_ generation by constructing genome-wide mutant library in rice (Lu et al., 2017; Meng et al., 2017). Given that the high editing efficacy in T_0_ generation, this set of CRISPR/Cas9 platform may have the potential to construct a genome-wide mutant library in rapeseed, which will further facilitate gene functional characterization and the later stage of application.

## Experimental procedures

### sgRNA expression module construction

Evidence shows that Arabidopsis U6-26 promoter is effective for driving the expression of sgRNAs in dicot plants (Li et al., 2007), We aimed to construct a sgRNA expression module driven by the AtU6-26. Based on searching Arabidopsis genome database (http://www.arabidopsis.org/), a 307-bp core promoter sequence was selected for fusing with sgRNA-scaffold. To make this sgRNA expression module compatible with the destination vector pYLCRISPR/Cas9P35S-N, the AtU6-26/sgRNA-scaffold was flanked with two BsaI restriction enzyme recognition sites as described previously (Ma et al., 2015). Then this sequence was synthesized and used as template in the following experiment.

### sgRNA design and CRISPR/Cas9 vector assembly

To simultaneously knockout all five rapeseed *SPL3* homoeologs, multiple alignment analysis was performed to find out high similarity DNA sequence that immediately upstream of NGG. Finally, a 22-bp target sequence located in the first exon region was chosen for CRISPR/Cas9 vector construction. The procedure for assembling sgRNA expression module followed the protocol reported (Ma et al., 2015). The PCR product of *BnSPL3* sgRNA expression module was purified and cloned into pYLCRISPR/Cas9P35S-N destination vector through golden gate assembly approach.

### Rapeseed transformation

After *BnSPL3* sgRNA expression module was assembled into pYLCRISPR/Cas9P35S-N vectors, the positive BnSPL3 sgRNA/ pYLCRISPR/Cas9P35S-N vector was introduced into *B. napus* cv. *ZhongShuang 6* (ZS6) through *Agrobacterium tumefaciens* (strain EHA105)-mediated transformation as described previously (Liu F. et al., 2014).

### Page-based mutation screening

Genomic DNA was extracted from transgenic rapeseed plants using the Plant Genomic DNA Kit (TIANGEN, China). The positive transgenic plants were identified by PCR amplification of *NPTII* gene. The genomic region flanking targeted mutation sites of SPL3A, SPL3B, and SPL3C were PCR-amplified using gene specific primers as shown below: SPL3A-FP: GAAAGGAAGCAAAGCAGAAGC, SPL3A-RP: CCTGACAAGTACTACTGCTG; SPL3B-FP: TATATCTTTCATGTGAGAGAGGA, SPL3B-RP: ACTCCACTATTACTGCTACTAC; SPL3C-FP: TGAGAGAGAGATAGTGGAATTC, SPL3C-RP: GGTACCACTGCTACTACTTG; SPL3D-FP: CTTCCATCTTTCATGTTTGAGAG, SPL3D-RP: TACCACTGCTACTACTTGTG; SPL3E-FP: CCATATTCTTACATGTGTGTGTG, SPL3E-RP: GACCTGACAAGCTCCAGC.

To make sure the quality of subsequent PAGE gel experiment, PCR product size ranged from 150 to 300 bp. The annealed PCR products were analyzed by 10% non-denaturing polyacrylamide gel electrophoresis (PAGE) gel.

### High-throughput sequencing analysis

Based on PAGE-based screening results, several PCR products from plants with obvious heteroduplex bands were chosen for constructing next generation sequencing libraries following the manufacture's protocol (Illumina TruSeq DNA Nano Library Prep Kit). Sequencing was carried out using 2 × 150 paired-end configuration (Illumina, San Diego, CA, USA). Data processing and statistical analysis was performed as described previously (Tang et al., 2017).

## Author contributions

CL, QH, HC, and BZ designed the experiments and wrote the paper. HC designed the sgRNA and construct. MH did the transformation and selection. WW and WC carried out the PAGE analysis. DM and HW did sequence analysis. FC helped with plant regeneration and maintenance. All of the authors read and approved the manuscript.

### Conflict of interest statement

The authors declare that the research was conducted in the absence of any commercial or financial relationships that could be construed as a potential conflict of interest.

## Supplementary material

Figure S1 PAGE-based identification of CRISPR/Cas9 induced mutation in *SPL3* genomic site **(A–D)** represent the PAGE analysis results of genomic sites *BnSPL3B, BnSPL3C, BnSPL3D*, and *BnSPL3E*, respectively. Red arrows indicate the examples of typical mismatched heteroduplex double-strand DNA bands.
