# Supplementary figures and images for: An Efficient CRISPR/Cas9 Platform for Rapidly Generating Simultaneous Mutagenesis of Multiple Gene Homoeologs in Allotetraploid Oilseed Rape
Source: Front Plant Sci. 2018 Apr 20;9:442. doi: 10.3389/fpls.2018.00442 (PMC5920024; doi:10.3389/fpls.2018.00442)

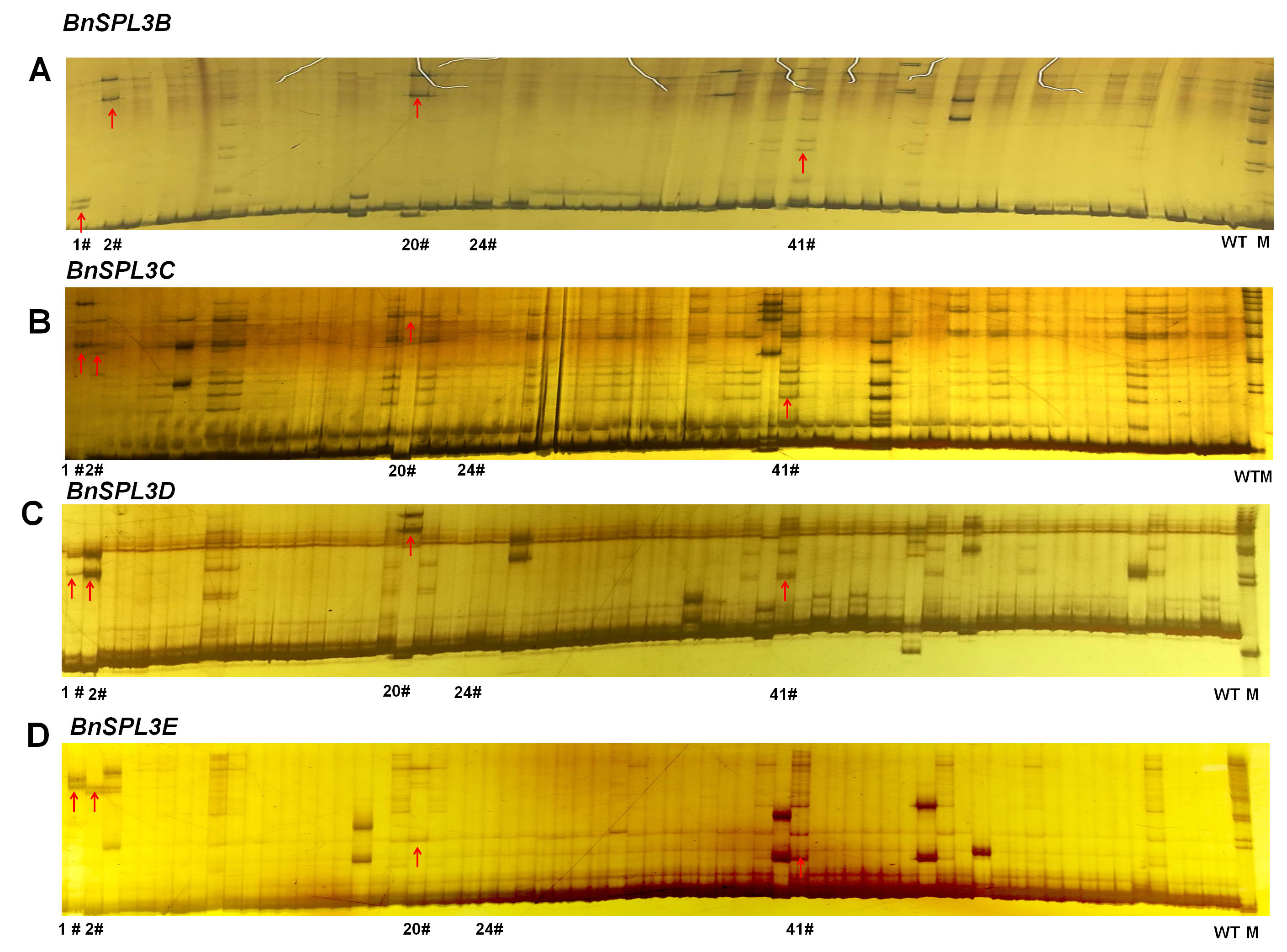

Supplement: Figure S1 — PAGE-based identification of CRISPR/Cas9 induced mutation in SPL3 genomic site (A–D) represent the PAGE analysis results of genomic sites BnSPL3B, BnSPL3C, BnSPL3D, and BnSPL3E, respectively. Red arrows indicate the examples of typical mismatched heteroduplex double-strand DNA bands. [file Image1.JPEG]
